# Upper Ureteric Stricture Secondary to Celiac Plexus Block Managed by Robotic Ureterocalicostomy

**DOI:** 10.1089/cren.2018.0082

**Published:** 2018-11-03

**Authors:** Deepak Ragoori, Mallikarjuna Chiruvella, Purnachandra Reddy Kondakindi, Mohd. Taif Bendigeri, Bhavatej Enganti, Syed. Md Ghouse

**Affiliations:** Department of Urology, Asian Institute of Nephrology & Urology, Hyderabad, Telangana, India.

**Keywords:** robotic ureterocalicostomy, iatrogenic ureteral stricture, complications of celiac plexus block

## Abstract

***Introduction:*** Ureterocalicostomy is a well-established procedure of choice for recurrent pelviureteric junction (PUJ) obstruction refractory to endoscopic management, failed pyeloplasty, completely intrarenal pelvis, and iatrogenic upper ureteral stricture with significant peripelvic fibrosis. Robotic ureterocalicostomy is the procedure of choice in such scenarios where meticulous dissection and accurate anastomotic suturing is required.

***Case Presentation:*** We report the case of an 18-year-old male, who underwent celiac plexus block for pain management of chronic calcific pancreatitis and presented with pain in the epigastric region and the right flank. A CT and subsequent nephrostogram revealed an upper ureteral defect (corrosive stricture) of ∼4 cm at the level of PUJ. Robotic ureterocalicostomy was performed. We discuss the clinical presentation, evaluation, and management along with literature review.

***Conclusion:*** Iatrogenic ureteral strictures are not uncommon in urological practice, but an upper ureteral stricture secondary to celiac plexus block is a rarity. Adequate evaluation and timely intervention by reconstructive surgery, robotic ureterocalicostomy in this case, yield satisfactory results.

## Introduction

Iatrogenic ureteral strictures are not uncommon in urological practice. Gynecological, colorectal, and pelvic surgeries, along with radiation, together comprise 75% cause of all ureteral strictures.^1^ It is a devastating complication, which if left untreated can result in urinoma, sepsis, renal failure, and eventually loss of renal unit.^2^ Surgical management is by a wide variety of procedures, starting from endoscopic management to reconstructive surgery depending on the location and length of the stricture.^3^ Celiac plexus block is a well-known entity in pain management of patients with chronic pancreatitis; however, it is rare to encounter urological complications secondary to celiac plexus block, which is what we have encountered and is discussed in this case report.

## Case Presentation

### History

An 18-year-old male was referred to us with history epigastric discomfort and right loin pain for a period of 3 weeks. The pain was localized, nonradiating, and dull aching in character. He had associated low-grade fever with multiple episodes of diarrhea and vomiting since 1 week. He is a known patient of chronic calcific pancreatitis, for which he underwent a celiac plexus block (10 mL of absolute ethyl alcohol) 2 months back. He had no comorbid conditions and no history of previous surgeries.

### Evaluation

**FIG. 1.**
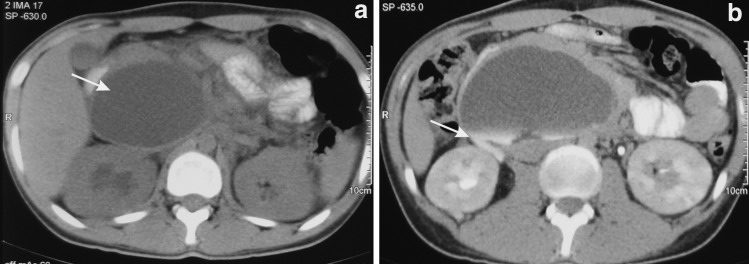
**(a)** Right hydronephrosis with a large well-defined collection in retropancreatic region extending along precaval and aortocaval region (urinoma). *Arrow* shows large urinoma which raises the concern of ureteric injury. **(b)** Contrast leak from the pelviureteric junction. *Arrow* confirms the source of urinary leak.

General examination revealed heart rate of 108 per minute and febrile with a temperature of 101°F. Abdominal examination revealed tenderness in the right hypochondriac quadrant and right loin. All other quadrants were normal with no palpable mass or tenderness. Laboratory investigations showed elevated total leukocyte count of 11,000 cu/mm and normal serum creatinine level of 1.01 mg/dL; rest of the parameters were within normal limits. Contrast-enhanced CT was done at the referral hospital and it showed right hydronephrosis with a large well-defined collection in retropancreatic region extending along precaval and aortocaval region (urinoma), with a contrast leak from the pelviureteric junction (PUJ) (Fig. 1).

**FIG. 2.**
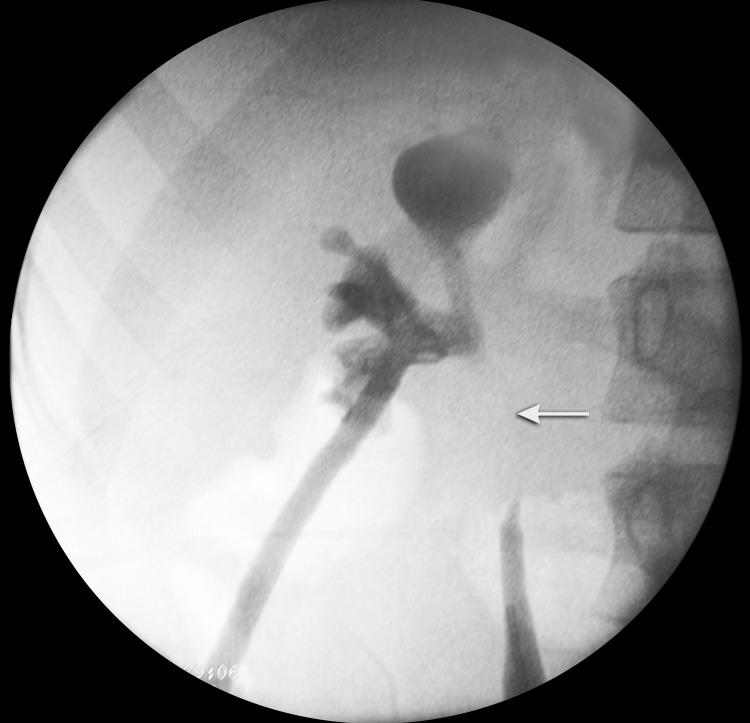
A simultaneous antegrade and retrograde pyelogram showing a long-segment (4 cm) upper ureteral defect. Abrupt contrast cut-off in renal pelvis and upper ureter highlighting the ureteric defect (*arrow*).

A retrograde pyelogram and attempt to secure a guidewire in to the right pelvicaliceal system failed with guidewire stopping ∼4 cm from PUJ. A 12F malecot catheter was placed as a right-sided percutaneous nephrostomy under ultrasound guidance. On follow-up, after 3 weeks, a repeat CT urogram was performed, which shows a complete resolution of urinoma. A simultaneous antegrade and retrograde pyelogram were done under anesthesia (Fig. 2). A long-segment ureteral defect was seen with an abrupt cutoff of the contrast 4 cm from PUJ on retrograde pyelogram and contrast seen within the renal pelvis on antegrade pyelogram.

### Intervention

**FIG. 3.**
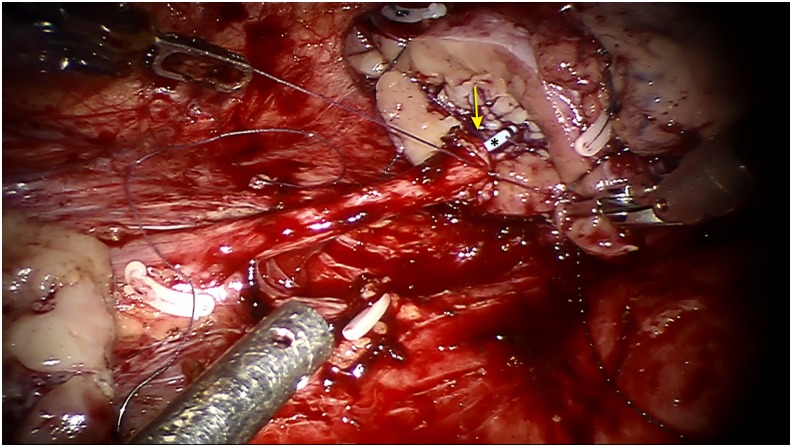
Robotic ureterocalicostomy being done with Double-J stent (*) in position. Site of ureterocalicostomy with Double-J stent across the anastomotic site (*arrow*).

Having assessed the patient and with a diagnosis of long segment upper ureteral stricture, we have decided to go ahead with robotic ureterocalicostomy. The procedure is done in standard left lateral position with three-port technique. After adequate colonic mobilization, ureteral mobilization was done only to see a fibroses upper ureter and a distorted renal pelvis. The upper end of ureter was transected just below the level of fibrosis. Subsequent dissection around hilum revealed a significant fibrosis. With a large ureteral defect to bridge and distorted renal pelvis, we have decided to go ahead with ureterocalicostomy. First, renal descensus was done to marginally cut down on the length of defect and then, after hilar clamping, lower polar parenchymal division was done to expose the lower calix. Hemostatic sutures were taken to control the parenchymal bleed and then, caliceal mucosal eversion was done. Parenchymal approximation was done on either side of the exposed calix. The dissected upper end of ureter was splayed and a tension-free and water-tight ureterocalicostomy was completed after placing a Double-J stent (Fig. 3).

### Follow-up

**FIG. 4.**
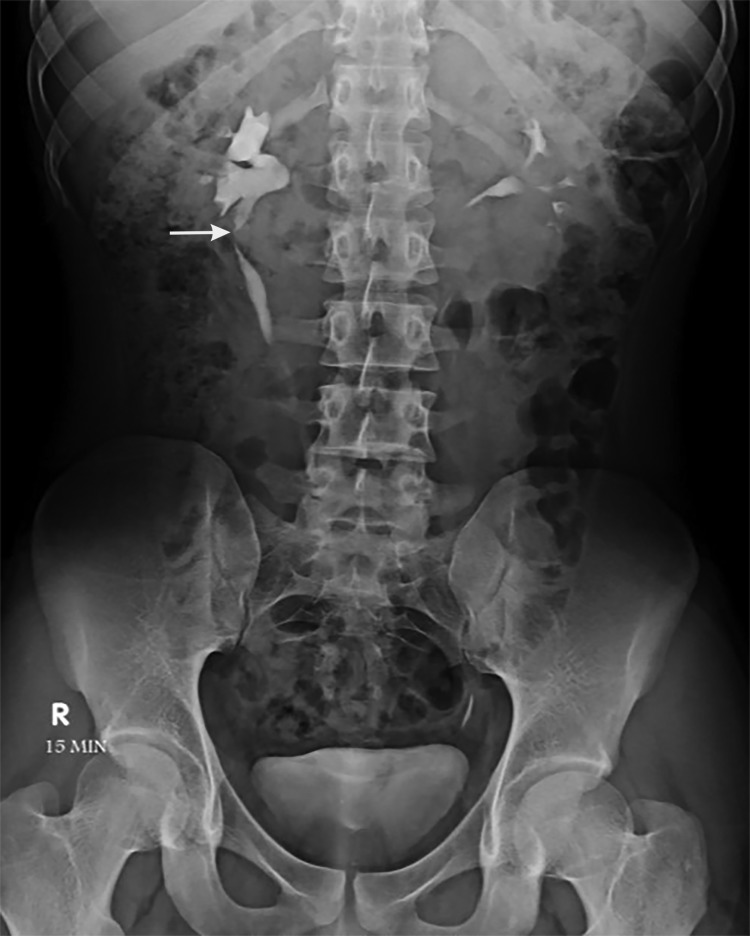
Intravenous pyelography done after 4 months, showing good drainage of contrast across the ureterocalicostomy site (*arrow*).

Postoperative period was uneventful and free of complications. Abdominal drain was removed on the fourth postoperative day. Patient was discharged on the eighth postoperative day. Double-J stent was removed after a duration of 4 weeks. Intravenous pyelography was done after 4 months (Fig. 4), which shows an intact ureterocalicostomy anastomosis and good drainage of contrast.

## Discussion

Iatrogenic ureteral injuries and strictures are relatively common complications of pelvic surgeries and radiation treatment. Although the distal third of the ureter is a frequent site of injury (91%), the middle and proximal third are rarely affected (7% and 2%, respectively)^3^

The management of ureteral injuries and strictures shows a broad range of therapeutic options from endoscopic management to complex reconstruction or even renal autotransplantation, and depends on the location, that is, proximal, mid, or distal ureter and the length and severity.^3^ Celiac plexus block is most commonly indicated in pain management of patients with intractable abdominal pain, pancreatic cancer, and occasionally also used in the treatment of pain from chronic pancreatitis, which helps in long-lasting improvement in pain and decreased narcotic usage in 70% to 90% of patients.^4^ Common complications are orthostatic hypotension and diarrhea. Less common complications include paraplegia, local anesthetic toxicity, spinal or epidural injection, aortic or vena cava puncture and bleeding, retroperitoneal hemorrhage, visceral organ injury, and pneumothorax. Literature search has not revealed any possible urological complications as discussed in this case, where injection of absolute alcohol for celiac plexus block has resulted in iatrogenic necrotic upper ureteral corrosive stricture. In this case, the complexity of the situation is worse because of the long ureteral segment loss.

Small ureteral defects (2–3 cm) of mid and upper ureter can be primarily repaired by ureteroureterostomy. However, the success of any treatment modality depends on the length of the ureteral stricture, the cause of the stenosis, and the location of the stricture.

The challenges we faced in a case like this with a corrosive stricture are an infrarenal pelvis, 4 cm upper ureteral defect, significant periureteric and hilar fibrosis, and a preexisting percutaneous nephrostomy tube.

According to the literature search, there are no data available on urological complications following celiac plexus block, in this case, an upper ureteral injury leading to urinoma and eventually leading to a ureteral stricture. We managed this case in a staged manner starting with a percutaneous nephrostomy and later a definitive surgery in form of robotic ureterocalicostomy.

## Conclusion

Celiac plexus block resulting in a urological complication, upper ureteral stricture in this case, has not been reported in the literature, to the best of our knowledge, hence a rarity. Corrosive ureteral strictures tend to have extensive periureteric fibrosis, which can be challenging during surgery and ureterocalicostomy, along with which it adds to the complexity and technically challenging scenario. In such situations, robotic surgery has got a role to play to perform meticulous dissection and a good anastomosis.

## Disclaimer

This case was presented as a video in SZUSICON at Wayanad, India, in August 2019, but not published in any journal.

## Abbreviations Used

CT: computed tomography
PUJ: pelviureteric junction
